# Impacts of feeding preweaned calves milk containing drug residues on the functional profile of the fecal microbiota

**DOI:** 10.1038/s41598-017-19021-2

**Published:** 2018-01-11

**Authors:** Richard Van Vleck Pereira, Laura M. Carroll, Svetlana Lima, Carla Foditsch, Julie D. Siler, Rodrigo Carvalho Bicalho, Lorin D. Warnick

**Affiliations:** 1 Department of Population Health and Reproduction, Cornell University, Ithaca, New York USA; 2 Department of Food Science, Cornell University, Ithaca, New York USA; 3 College of Veterinary Medicine, University of California Davis, Davis, CA USA; 4 Department of Population Medicine and Diagnostic Sciences, College of Veterinary Medicine, Cornell University, Ithaca, NY United States of America

## Abstract

Feeding drug residue-containing milk to calves is common worldwide and no information is currently available on the impact on the functional profile of the fecal microbiota. Our objective was to characterize the functional profile of the fecal microbiota of preweaned dairy calves fed raw milk with residual concentrations of antimicrobials commonly found in waste milk from birth to weaning. Calves were assigned to a controlled feeding trial being fed milk with no drug residues or milk with antibiotic residues. Fecal samples collected from each calf once a week starting at birth, prior to the first feeding in the trial, until 6 weeks of age. Antibiotic residues resulted in a significant difference in relative abundance of microbial cell functions, especially with genes linked with stress response, regulation and cell signaling, and nitrogen metabolism. These changes could directly impacts selection and dissemination of virulence and antimicrobial. Our data also identified a strong association between age in weeks and abundance of Resistance to Antibiotics and Toxic Compounds. Findings from this study support the hypothesis that drug residues, even at very low concentrations, impact the gut microbiota of calves and result in changes in the functional profile of microbial populations.

## Introduction

Feeding waste milk, the non-saleable milk from cows with milk withhold because of treatment with therapeutics or cows with high somatic cell counts, to dairy calves is a common practice in the United States (~33% of dairy farms), as well as in other countries^1,2^. However, there is growing concern that this practice can lead to antimicrobial-resistant (AMR) bacteria, which is reflected in the recent release of a scientific opinion by the European Food Safety Authority (EFSA) Panel on Biological Hazards (BIOHAZ)^3^, which concludes that the practice of feeding milk containing drug residues to calves has a high risk for increasing fecal shedding of AMR bacteria by calves.

The use of antimicrobial drugs for treatment, prevention or control of disease in livestock has, in recent years, been under scrutiny by public health, food safety, and regulatory perspectives due to concerns with potential for development of antimicrobial resistance^4^. Selection of resistant bacteria has generally been assumed to occur at concentrations between the minimal inhibitory concentration (MIC) of the susceptible wild type population and that of the resistant bacteria, and concentrations below the MIC of the susceptible population were considered to not inhibit growth of the susceptible bacteria and, therefore, were unable to cause selection pressure^5,6^. Nevertheless, studies using highly sensitive competition experiments have shown that selection of resistant bacteria can occur at extremely low antibiotic concentrations, selecting for resistant bacteria with compensatory mutations that counterbalance the decreased fitness cost caused by resistance^7–9^.

Exposure of bacteria to antimicrobial drugs at sub-MIC concentrations has been shown to stimulate mutagenesis and recombination, leading to bacterial adaptation to various stresses, including antibiotic pressure^10,11^. A recent study by our group has also shown that calves receiving milk containing residual concentrations of ampicillin, ceftiofur, penicillin, and oxytetracycline from birth to weaning can result in clear discriminate gut microbial communities^12,13^. However no study has evaluated the impacts of this practice on the functional fecal microbiota of preweaned calves. Therefore, the objective of this study was to characterize the functional profile of the fecal microbiota of preweaned dairy calves fed raw milk with residual concentrations of ampicillin, ceftiofur, penicillin, and oxytetracycline from birth to weaning.

## Results

### Sequencing Data

Shotgun sequencing was used to compare gene functional distribution from 56 fecal samples collected from 14 dairy calves at pre-treatment (S0), and at weeks one (S1), three (S3) and six (S6) after the beginning of the study. One sample from a calf in the DR group collected at S0 was not included in the study because it rendered a very low number of reads (total of 389 sequences read). Sequencing data for the remaining samples is available in Supplementary Table S1. Our study had a total of 212,571,492 sequences read, with a mean sequence length of 268 bp (95% CI: 266–270 bp).

### Effect of Drug Residues in Milk on Microbial Function

**Figure 1:**
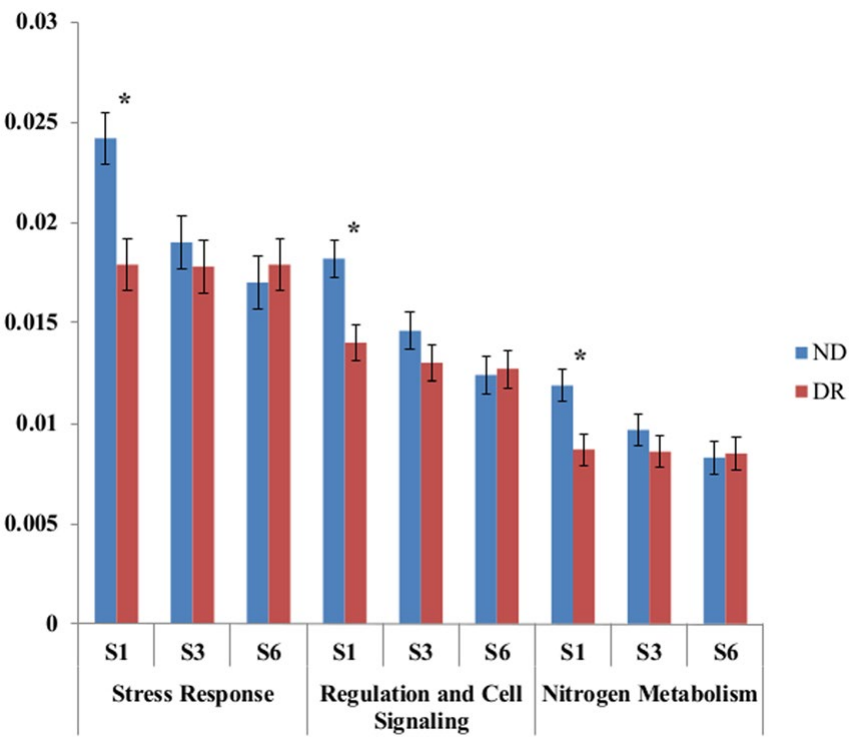
Abundance of “stress response”, “regulation and cell signaling”, and “nitrogen metabolism”. Sequences in fecal samples collected from control calves (NR) and calves feed milk containing low concentrations of drug residues (DR) for samples collected as 1 (S1), 3 (S3) and 6 (S6) weeks after beginning of trial. Values are expressed as a percentage of the total sample sequences assigned function. The symbol * indicates significant differences (*P*-value ≤ 0.05). Error bars represent 95% confidence interval of the least square mean.

**Figure 2:**
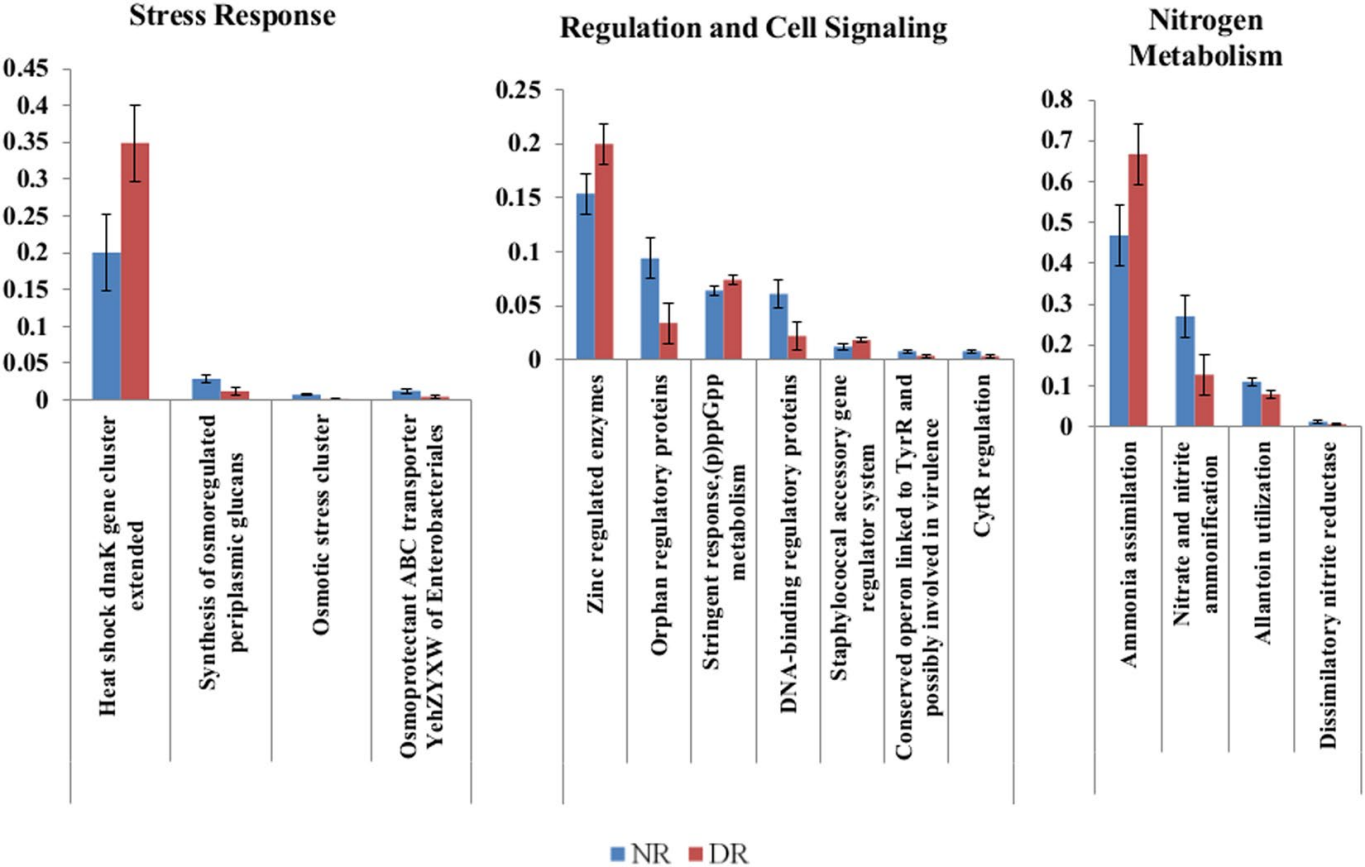
Normalized abundance of gene functions at level 3 for “stress response”, “regulation and cell signaling”, and “nitrogen metabolism”. Results are for samples collected at S1. Only functions for which a significant difference between DR and NR samples was detected were included in the table (*P* < 0.05). Error bars are standard error of the mean.

The distribution of microbial cell function in calf feces by control and treatment group is available in Table 1. When comparing microbial function between treatment groups (data from weeks S1, S3 and S6 clustered together), calves receiving drug residues in the milk had a significantly higher abundance of genes for “DNA Metabolism” and “Cofactors, Vitamins, Prosthetic Groups, Pigments”, although this difference was not significant when comparing treatment group by sampling week. Samples from calves not receiving drug residues in the milk had significantly higher abundances for “Motility and Chemotaxis”, “Nitrogen Metabolism”, “Regulation and Cell Signaling”, and “Stress Response”, with a significant difference also observed between treatment groups by week of sample collection for “Nitrogen Metabolism”, “Regulation and Cell Signaling”, and “Stress Response”. Evaluation of these three function categories per week for each treatment group revealed that the significant difference between these groups was only observed at week 1 (S1) (Fig. 1). Further analysis at level 3 for these three microbial functions were conducted for samples collected at S1, and functions for which a significant difference was observed between treatment groups are available in Fig. 2.

**Table 1:** Distribution of microbial cell function in calf feces by control (NR) and treatment group (DR). Least square means is accumulated by treatment group for samples collected at week 1, 3 and 6. *P*-value is relative to significant differences in cell functions between treatment groups and for different sampling times.

Functional Category	NR^1^	DR^2^	SEM^3^	*P*-Value	
				Tx Group^4^	Tx Group x Week^5^
Amino Acids and Derivatives	0.071	0.072	0.0004	0.42	0.43
Carbohydrates	0.121	0.118	0.003	0.22	0.28
Cell Division and Cell Cycle	0.016	0.018	0.0004	0.06	0.23
Cell Wall and Capsule	0.044	0.042	0.0006	0.11	0.68
Clustering-based subsystems	0.161	0.167	0.003	0.07	0.67
Cofactors, Vitamins, Prosthetic Groups, Pigments	0.056	**0.059**	0.0006	**0.02**	0.34
DNA Metabolism	0.05	**0.052**	0.001	**0.04**	0.22
Dormancy and Sporulation	0.0031	0.0033	0.00021	0.26	0.15
Fatty Acids, Lipids, and Isoprenoids	0.021	0.022	0.0004	0.65	0.97
Iron acquisition and metabolism	0.01	0.0091	0.002	0.46	0.95
Membrane Transport	0.0403	0.0356	0.002	0.11	0.20
Metabolism of Aromatic Compounds	0.0069	0.0068	0.00035	0.87	0.21
Miscellaneous	0.083	0.089	0.002	0.06	0.46
Motility and Chemotaxis	**0.0052**	0.0042	0.0003	**0.04**	0.11
Nitrogen Metabolism	0.0099	0.0086	0.00059	**0.04**	>**0.05**
Nucleosides and Nucleotides	0.034	0.0362	0.001	0.14	0.91
Phages, Prophages, Transposable elements, Plasmids	**0.024**	0.0205	0.001	**0.04**	0.48
Phosphorus Metabolism	0.0095	0.0098	0.00035	0.24	0.23
Photosynthesis	0.00037	0.00036	0.00008	0.85	0.76
Potassium metabolism	0.0054	0.0051	0.0005	0.33	0.90
Protein Metabolism	0.079	0.082	0.002	0.22	0.20
Regulation and Cell signaling	0.015	0.013	0.0006	**0.03**	**0.02**
Respiration	0.018	0.014	0.0014	0.05	0.57
RNA Metabolism	0.044	0.046	0.001	0.17	0.24
Secondary Metabolism	0.0044	0.0042	0.00019	0.41	0.62
Stress Response	0.02	0.017	0.0009	**0.03**	**0.02**
Sulfur Metabolism	0.013	0.013	0.0013	0.42	0.65
Virulence, Disease and Defense	0.025	0.023	0.001	0.16	0.99

^1^Calves fed raw milk without the addition of sub-MICs of antimicrobial drugs; ^2^Calves fed raw milk with the addition of sub-MICs of ceftiofur, penicillin, ampicillin, and oxytetracycline from birth to 6 weeks of age; ^3^Standard error of the mean; ^4^*P*-value comparing function relative abundance between treatments groups (NR vs DR) for the mixed linear regressions; ^5^*P*-value comparing function relative abundance interaction between treatment groups (NR vs DR) and week samples was collected (1, 3 and 6) for the mixed linear regression.

**Figure 3:**
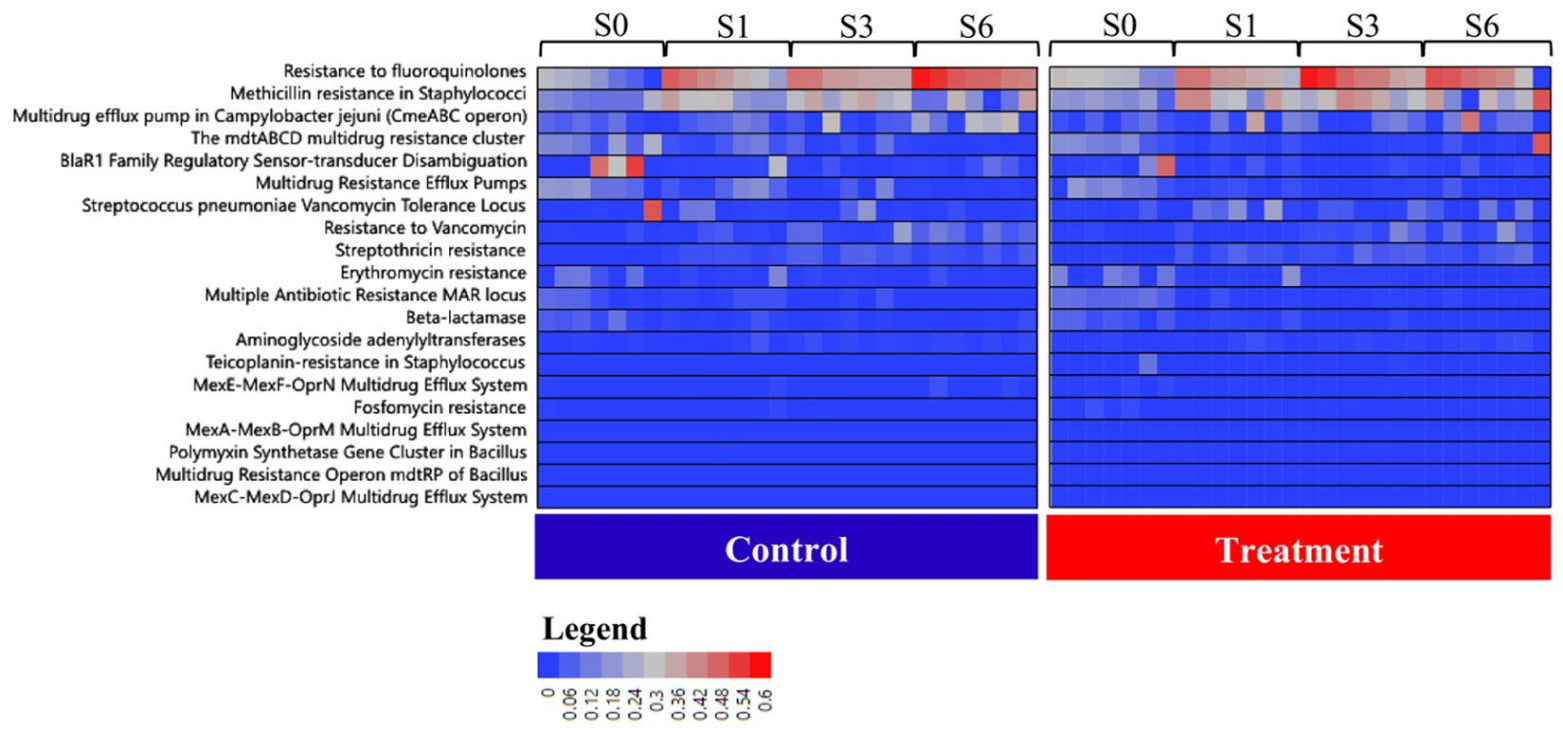
Heatmap displaying the distribution of the top 20 resistance to antibiotics and toxic compounds (RATC) functions. Results for samples collected at weeks S0, S1, S3 and S6 by treatment group for each individual fecal sample. Blue to red scale represents relative abundance for each function.

**Figure 4:**
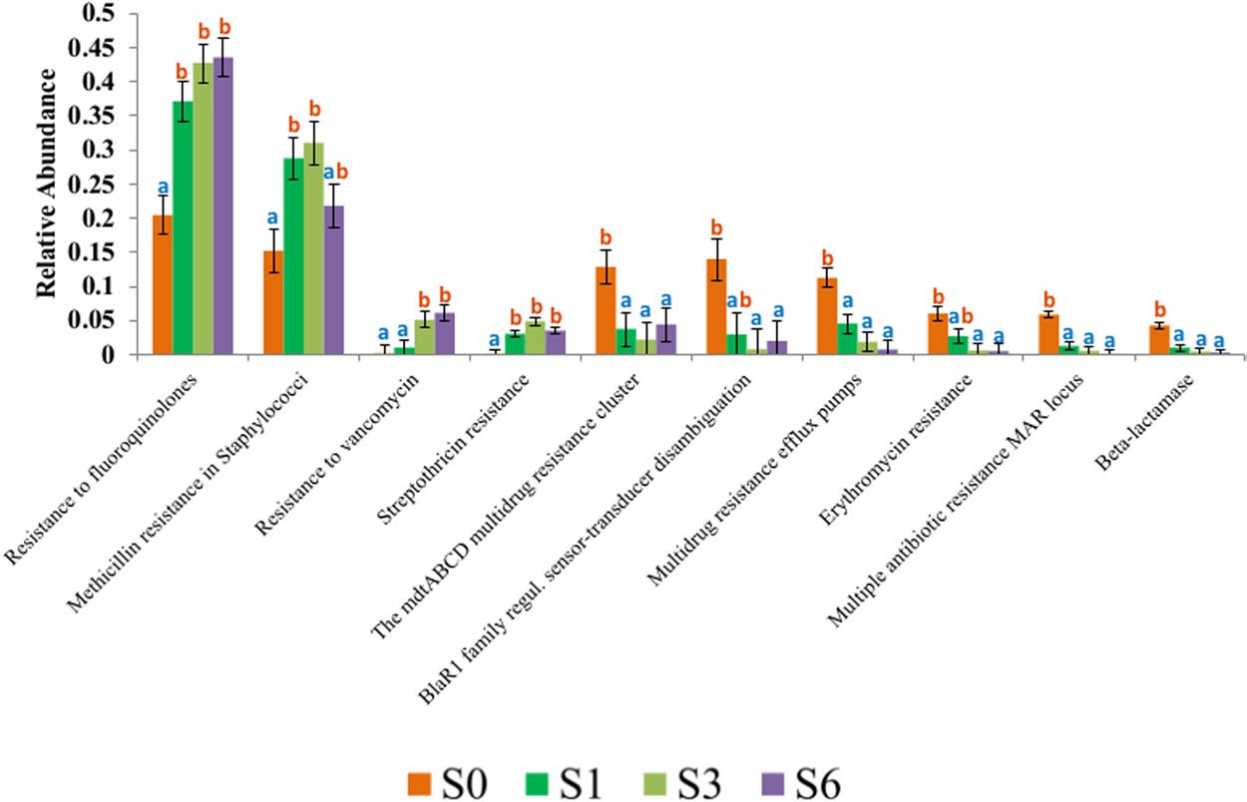
Distribution of the resistance to antibiotics and toxic compounds (RATC) functions that had significantly different relative abundance at different weeks. Results for samples collected at weeks S0, S1, S3 and S6. Different letters indicate time points that were significantly different based on pairwise analysis for each function.

**Figure 5:**
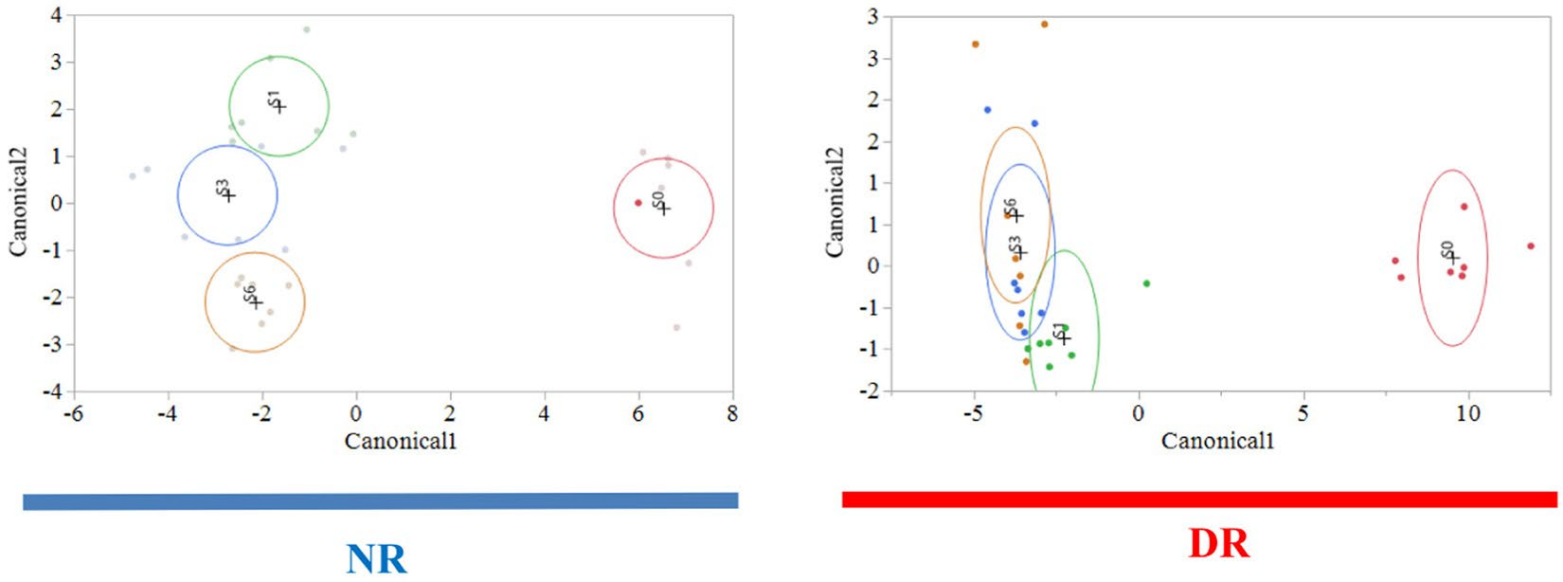
Discriminant analyses of fecal samples based on RATC function of microbiota for each treatment group by sampling week. Differences in in RATC profiles for each sampling week (pre-treatment, S0 = red dots; week 1, S1 = green dots; week 3, S3 = blue dots; week 6, S6 = orange dots) are illustrated by Canonicals 1 and 2. A circle indicates the 95% confidence region to contain the true mean of the group, and a plus symbol indicates the center (centroid) of each group.

**Figure 6:**
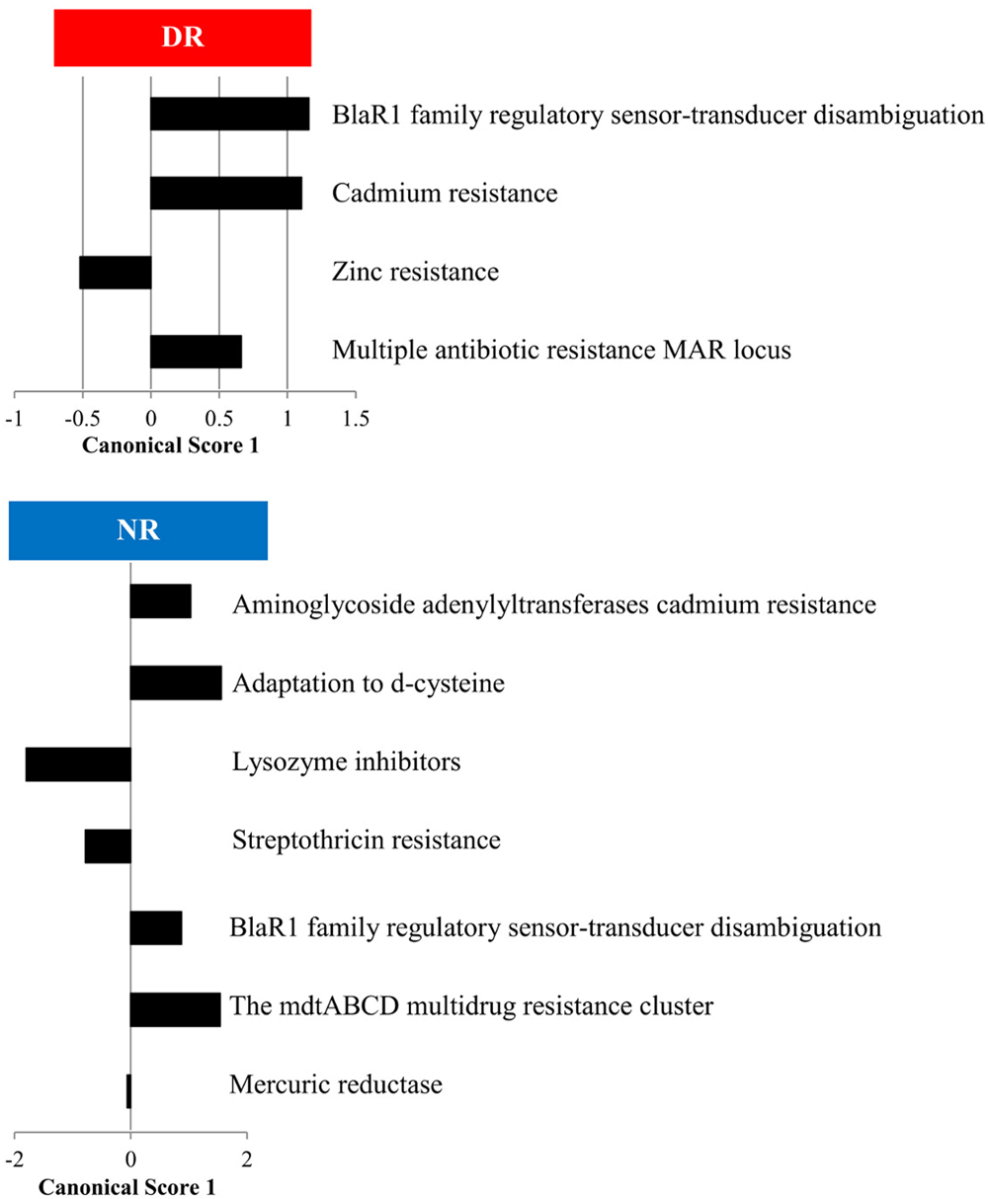
Canonical scores for RATC profile that were found to be significant for the discriminant analysis displayed in Fig. 4.

The distribution of Resistance to Antibiotics and Toxin Compounds (RATC) by treatment group for each sampling time point (S0, S1, S3 and S6) is shown using a heatmap in Fig. 3. An evaluation of the distribution of RATC by sampling time point (S0, S1, S3 and S6) is displayed in Fig. 4. No significant abundance of RATC genes between treatment groups for each sampling point was observed, although a discriminative analysis for RATC indicated that there are genes within this category that have a significantly different distribution according to which week these samples were collected (Fig. 5). Canonical values for this analysis are displayed in Fig. 6.

### Phylogenetic Analysis

**Figure 7:**
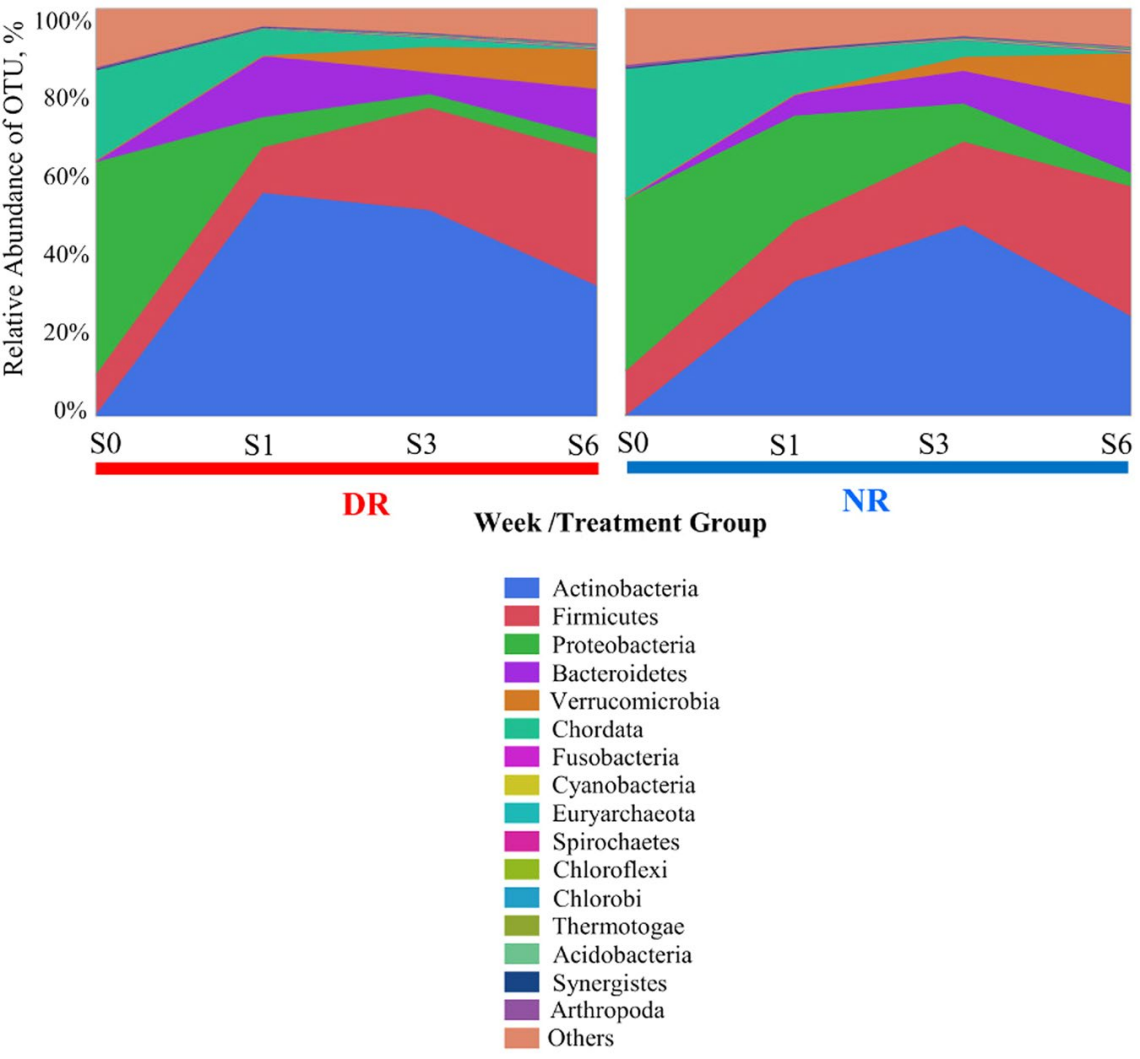
Average percentage of the 16 most prevalent phyla for each treatment group by week of sampling.

**Figure 8:**
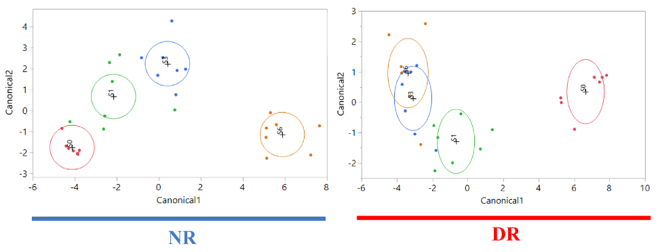
Discriminant analyses of fecal sample microbiome for each treatment group (NR and DR) by week. Differences in the fecal microbial profiles for each sampling week (S0 = red dots, S1 = green dots, S3 = blue dots, S6 = orange dots) are illustrated by Canonicals 1, and 2. An ellipse indicates the 95% confidence region to contain the true mean of the group, and a plus symbol indicates the center (centroid) of each group.

The relative abundance of the 16 most common phyla by treatment group (DR and ND) is displayed in Fig. 7. Microbiota characterization and analysis using sequencing of the 16S rRNA gene of these samples using the Illumina MiSeq platform is available elsewhere on a previous publication^12^. A discriminant analysis for the microbiota over time in weeks for each treatment group is shown in Fig. 8. Shannon diversity was not significantly different between treatment groups at different sampling points or between different sampling points alone. Mean Shannon diversity index at the species level was 5.95 (95% C.I.: 5.90–6.01).

## Discussion

Feeding preweaned calves milk containing drug residues resulted in a significant difference in relative abundance of microbial functional genes. Evaluation of the impacts of treatment with milk containing drug residues revealed a significant difference in the prevalence of 6 different functions (Supplementary Table S1). A more in-depth analysis for each sampling week revealed that the sampling point where the greatest difference was observed was one week after the beginning of the treatment (S1), resulting in a significantly lower abundance of microbial genes linked with “Stress Response”, “Regulation and Cell Signaling”, and “Nitrogen Metabolism”.

Further evaluation of Stress Response at S1 revealed a few functions present at a greater abundance within that functional category for NR group, namely, “Osmotic Stress”, “Desiccation Stress” at level 2, and “Osmoprotectant ABC Transporter *yehZYXM* of Enterobacteriaceales”, “Osmotic Stress Cluster”, and “Synthesis of Osmoregulated Periplasmic Glucans” at level 3. Osmoregulation is known to be fundamental for living cells, in order to avoid intolerable water flow rates through the plasma membrane, which would result in cell death^14^. A higher abundance of these functions related to osmoregulation in NR could potentially translate into a greater ability of the enteric microbiota in the NR group to withstand osmotic pressures, such as that caused by desiccation, or a higher concentration of sugars or salts. Osmotic stress in the calf gut can be caused by a number of environmental factors, including diet. Although calves in the study were fed whole milk, another common contribution to the calf diet employed by dairy farms is milk replacer, which is known to have a higher osmolality, and which may intermittently be a source of feed according to availability of waste milk on the farm^15^.

Additionally, “Heat Shock” stress genes were present in greater abundance in DR group, with “Heat Shock *dnaK* Gene Cluster Extended” being observed at greater abundance at level 3. Heat shock genes are primarily responsible for the production of heat shock proteins (HSPs), which are known to have an important role in cellular functions, such as protein trafficking, receptor maturation, and signal transduction^16,17^. HSPs include chaperones and proteases that are considered vital for overcoming changes that involve protein denaturation. Exposure of commensal bacteria, such as *Escherichia coli*, to antibiotics has been shown to induce heat shock protein expression^18^. The *dnaK* gene cluster is formed by different genes, including *dnaK*, *dnaJ*, and *grpE*, which are related to the production of proteins shown to be involved in the negative regulation of the heat shock response by affecting the synthesis and activity, as well as the stability of sigma factor 32 (σ^32^)^19^. σ^32^ is the transcription factor responsible for the recognition of heat shock promoters, and has been shown to regulate heat shock response in gram-negative enteric bacteria such as *E. coli*, so genes in the “*DnaK* gene cluster” are considered the actual heat stress sensors^20,21^. Besides their roles in protecting cellular proteins from environmental insults and in maintaining cellular homeostasis, some HSPs have been shown to increase bacterial virulence. One study indicated *DnaK* participated in the expression of virulence genes in *Listeria monocytogenes* (*flaA* and *lmaB* genes) and *Vibrio cholera* (*ToxR* gene)^22,23^.

A deeper look at microbial genes associated with regulation and cell signaling revealed that at S1 the microbiota in the DR group had a higher relative abundance of genes associated to “Regulation and Cell Signaling”. Some functions observed in greater abundance in the DR group were “zinc regulated enzymes”, “stringent response”, “(p)ppGpp metabolism”, and “Staphylococcal accessory gene regulator system”. Zinc is an important trace mineral utilized during the growth in many microorganisms, and regulation of zinc plays a key role in microbial composition: low quantities of zinc may not support microbial growth, while concentrations that are too high may be toxic^24^. A study evaluating the effect of zinc competition among intestinal microbiota in chickens showed that *Campylobacter jejuni* that lacked a high-affinity zinc-binding protein (ZnuA) were not able to compete for zinc in the enteric microbial environment, limiting the ability to successfully colonize the intestinal tract^24^. Another study that fed piglets a source of zinc that resulted in a lower availability to the microbiota resulted in a significantly lower microbiota richness and diversity when compared to the control diet with higher availability of zinc^25^. This indicated that zinc has a direct impact on microbial composition, selecting for microbes that were able to adapt to lower zinc concentrations. Piglets in the treatment group also had a lower prevalence of diarrhea, which could have occurred due to a shift in the prevalence of highly virulent pathogenic microbes in the intestinal microbiota.

The stringent response is a regulatory mechanism mediated by two related alarmone nucleotides, guanosine tetraphosphate and guanosine pentaphosphate, collectively referred to as (p)ppGpp^26^. The primary target of (p)ppGpp is transcription, and its importance is evident during acute survival situations, including stresses such as nutrient deprivation or heat shock^27^. The stringent response is also known to be important for bacterial virulence and persistence in the environment (e.g. resistance to antimicrobials) for a variety of taxonomic divisions. Exposure of microbes to stressful conditions is believed to result in higher ppGpp levels, as would be the case for microbes in DR, which were exposed to low levels of drug residues for an extended period of time^28–30^.

Accessory genes regulators (AGR) have been shown to have a crucial role in the development of skin infection in community-associated methicillin-resistant Staphylococcus aureus (CA-MRSA), leading to remarkably strong expression of toxins and exoenzymes, increased expression of methicillin resistance genes, upregulation of fibrinogen-binding proteins, and increased capacity to bind fibrinogen^31–33^. Although AGR have been shown to increase *Staphylococcus* virulence, including by lysing neutrophils through regulation of toxins, there is evidence of a trade-off between loss of accessory genes regulators and antimicrobial resistance^34^. Furthermore, carriage of defective AGR has been linked to exposure of *Staphylococcus* to antimicrobials such as fluoroquinolones or beta-lactams; this may be particularly relevant in cases where bacteria face extended exposure to drug residues present in milk^35^. The current study did not evaluate the functionality of AGR genes, making it difficult to estimate the potential impacts of a higher abundance of AGR genes in the microbiota of DR calves compared to NR calves.

Many of the functions observed in higher abundance in NR calves at S1 are related to regulation of microbial transcription. In *Escherichia coli*, CytR-encoded repressor protein (CytR) has been shown to control the expression of several genes involved in nucleoside and deoxynucleoside uptake and metabolism^36^. In *Vibrio cholera*, CytR not only has a role in nucleoside catabolism but also as a biofilm repressor, specifically, controlling the synthesis of the *Vibrio cholerae* exopolysaccharide^37^. The higher abundance of CytR in NR microbiota was not surprising considering that it is a DNA-binding protein, which was also observed at a higher abundance in the microbiota of NR calves^38^. The Operon linked to TyrR, a transcriptional regulator that controls the metabolism of aromatic amino acids in *Escherichia coli*, was also observed in higher abundance in NR^39^. Due to its metabolic role, TyrR also has an important virulence role for pathogenic *Yersinia pestis*^40^. NR calves also had a higher abundance of “Orphan Regulated Enzymes” at S1. Because this term comprises enzymes of unknown function, it is difficult to derive any potential biological meaning from this functional group’s difference in abundance between NR and DR microbiota^41^.

Ammonia is the preferred source of nitrogen for the growth of enteric bacteria, and is incorporated into glutamate and glutamine, providing nitrogen for the synthesis of most of the other amino acids^42,43^. The preference for ammonia is believed to be related to a fast growth rate when compared to alternative nitrogen sources such as amino acids, as has been shown in *E. coli*^44^. Therefore, as long as this nutrient resource is available, the higher abundance of genes with this function in the microbiota of DR calves could potentially translate into an enteric microbiota capable of growing at a faster rate after disturbances to the microbiota.

The dissimilatory nitrate reduction to ammonium occurs in two phases: first nitrate is reduced to nitrite, and then nitrite is reduced to ammonium^45^. Genes associated with this process were observed in higher abundance in the microbiota of NR calves. A previous study has shown that, in the presence of relatively high physiological nitrate concentrations, two common bacterial species colonizing the human intestine (*E. coli* and *Lactobacillus plantarum*) generate nitrite and, subsequently, ammonia in an oxygen-dependent fashion^46^. There is still limited information about the importance of this pathway *in vivo*, and further studies are needed to fully interpret the potential impacts of having a higher abundance of genes associated with this function^46^.

Allantoin is a naturally occurring compound and a major metabolic intermediate in most living organisms, including bacteria. It is produced in the degradation pathway of purine nucleobases by action of a urate oxidase enzyme on uric acid^47^. In bacteria, purines are used as secondary sources of nitrogen under conditions where the preferred sources of nitrogen (ammonia or glutamate) are not available^48^. The higher abundance of genes associated to this function in NR calves could indicate an adaptation of the microbiota to lower ammonia concentrations, which may provide this microbiota with the ability to better adapt and compete in this environment when compared to the microbiota of DR calves.

Although no significant difference in relative abundance between treatment groups by sampling time point was observed for RATC, samples from NR calves had a more differentiated RATC functional profile when compared to samples from DR calves (Fig. 5). This suggests that drug residues exerted a selective pressure that impacted the selection and distribution of genetic functions in RACT, resulting in a transition to a microbial RATC function that is more similar over different sampling time points in DR when compared to NR calves. The lower impact of diversity on shaping the distribution of RATC genes can also be seen in the canonical score for this discriminative analysis (Fig. 6), which shows that only 4 functional categories in DR, when compared to 7 functional categories in NR, had a significant positive or negative effect on the differentiation of the RATC function distribution over time.

Greater antimicrobial resistance in younger calves has previously been observed in multiple culture-based studies evaluating phenotypic resistance of commensal enteric bacteria in preweaned calves^49,50^. A lack of developed intestinal microflora in young calves has been suggested as a factor in higher colonization of younger calves by drug resistant bacteria that may have a higher fitness cost^49^. Fitness cost is a measure of the ability of an individual microbe to survive and reproduce, and a mechanism that could confer drug resistance has been shown to potentially result in a high biological cost that could reduce survivability under conditions that do not provide a selective advantage (e.g. antibiotic-free environments). A degree of protection against colonization by bacteria with a higher fitness cost, such as antimicrobial-resistant bacteria and pathogenic enteric bacteria, has been suggested to follow as the calves’ indigenous microflora matures, resulting in a commensal-microbiota-mediated resistance to colonization^51,52^. However, there are a lack information of longitudinally studies evaluating the fecal microbiota of preweaned calves, as well as antimicrobial resistance genomic profiles. To account for this, we clustered the data from the two treatment groups to illustrate changes in RATC profiles over time on functions observed to be significantly different at different weeks of life (Fig. 4).

Our study strongly supports this theory, as demonstrated by the significant difference by week observed in the microbiome composition of newborn calves (Fig. 7), indicating a significant change over time in the major players participating in the microbial gut composition in calves as they age. This is in agreement with the findings of a previously published study using samples from this clinical trial where weekly fecal samples were evaluated for microbial composition by sequencing of the 16 S rRNA genes^12^. Furthermore, these findings support the potential relevance of future studies focusing on using approaches to support an early maturation of the microbiota in preweaned calves to help protect them from invasive pathogens, as well as create an unfavorable environment for harboring antimicrobial resistant microbes.

When evaluating RATC functions between sampling weeks alone, samples from S0 were predominantly observed to be significantly different from samples collected at S1, S3 and S6, with a few exceptions (Fig. 4). Only “resistance to fluoroquinolones” and “streptothricin resistance” were present at a significantly lower relative abundance at S0 when compared to the later three sampling points. “Resistance to vancomycin” was also significantly higher abundances in S3 and S6 when compared to S0 and S1. Although most RATC functions decrease over time (from S0 to S6), an increase in resistance to vancomycin (VAN) and fluoroquinolones (FLQ) is of great relevance, as these are two drugs of critical importance for treating disease caused by gram positive bacteria such as *Enterococcus* and *Staphylococcus aureus*, as well as diseases caused by gram negative pathogens such as *E. coli* and *Salmonella*^53,54^.

The higher prevalence of microbes associated with resistance to FLQ in a more competitive microbial environment could be explained by a low fitness cost related to some FLQ resistance mechanisms. Although this is not always the case and resistance to FLQ can be followed by a high fitness cost^55^, studies with *Salmonella enterica* serovar Typhi and *Campylobacter jejuni*, have shown that FLQ resistance-conferring mutations in the *gyrA* gene can have a positive influence on fitness: mutant strains are not only FLQ resistant, but have the ability to out-compete wild type strains in drug-free competitive experiments^56–58^. For VAN resistance, fitness cost has also supported dissemination of resistant strains^59^. As an example, VAN resistance in *Enterococcus spp*. (VanB-type) was shown to have tight regulation of VAN resistance expression^60^. When VAN is present, the VAN resistance mechanism is activated and results in a high biological cost. However when VAN is absent in the environment, the biological cost of this resistance mechanism is minimal.

RATC functions that decreased over time in the microbiota include three functions that could confer phenotypic multidrug resistance (MDR) through MDR efflux pumps (*mdtABCD* MDR cluster, MDR efflux pumps and multiple antibiotic resistance (MAR) locus)^61,62^, as well as two functions associated with resistance to beta-lactam (BlaR1 and Beta-lactamase) and macrolide drugs (erythromycin resistance). MDR efflux pumps have been associated with a high fitness cost, which would support the observed decrease in their prevalence in the microbiota as the microbiota matures into a more competitive environment^63^. As an example, overexpression of MDR efflux pumps in *Stenotrophomonas maltophilia*, an aerobic gram-negative bacterium, was associated with decreased fitness resulting in an impairment of bacterial physiology^64^. Similarly, macrolides and beta-lactam resistance mechanisms have frequently been linked with higher fitness costs, reducing their dissemination in highly competitive environments without a selective advantage from antimicrobial drugs^65–68^.

## Conclusion

Feeding preweaned calves milk containing drug residues resulted in a significant difference in relative abundance of genes linked to stress response, regulation and cell signaling, and nitrogen metabolism. Difference in abundance to these functions could have the potential to directly affect the resilience of the microbiota to adapt to environmental changes, such as changes in feed, which could have direct impacts on selection and dissemination of virulence and antimicrobial resistance in the microbiota. Drug residues exerted impacts on the selection and distribution of genetic functions in RACT, resulting in a transition to a microbial RATC function more similar over different sampling time points in DR when compared to NR calves. When compared to younger calves, fecal microbiota of older calves was linked with a higher abundance of functions associated with resistance to vancomycin and fluoroquinolones, two important drugs in human medicine.

## Methods

### Ethics statement

Fecal samples were collected from calves (*Bos taurus*) that were housed on Cornell University facilities. The research protocol was reviewed and approved by the Institutional Animal Care and Use Committee of Cornell University (Protocol number: 2012–0090). All experiments were performed in accordance with relevant guidelines and regulations.

### Study design and sample collection

Randomized non-blinded controlled feeding trials were conducted at the College of Veterinary Medicine, Cornell University (Ithaca, NY, USA) from June 2013 to March 2014. Three feeding trials were completed with a total of 10 male calves in each trial, with 5 calves belonging to each treatment group. All thirty calves enrolled in the trials were purchased from a local dairy farm and enrolled in the study on their date of birth. Control calves (n = 15) were fed raw milk without the addition of antimicrobial drugs (**NR**), and test calves (n = 15) were fed raw milk with the addition of low concentrations of ceftiofur, penicillin, ampicillin and oxytetracycline (**DR**). All calves were bucket fed one gallon of raw whole milk twice a day from birth to 6 weeks of age.

Antimicrobial stock solutions used to spike raw saleable milk fed to calves were prepared one week prior to each calf trial. Stocks were prepared by diluting powdered drugs in distilled water to a concentration of 100 μg/mL for ampicillin sodium salt, 1,000 μg/mL for ceftiofur sodium, 50 μg/mL for penicillin G sodium, and 3,000 μg/mL for oxytetracycline hydrochloride. The final concentration of each antimicrobial drug in the milk fed to DR calves was calculated to be: 0.01 μg/ml of ampicillin sodium, 0.1 μg/ml of ceftiofur sodium, 0.005 μg/ml of penicillin G sodium, and 0.3 μg/ml of oxytetracycline hydrochloride. Details on calf housing, feeding and management are described in detail a previously published article^69^. No significance difference (p = 0.97) was observed on average daily weight gain between calves in DR group (1.38 lbs, 95% CI 1.06–1.70) and NR group (1.38 lbs, 1.06–1.70), or on the average daily milk consumption during the trial (DR = 7.70 L, 95% CI 7.4–8.0; NR = 7.73 L, 95% CI 7.4–8.0), reducing the likelihood that feed intake was as a factor resulting in difference in fecal microbiota composition and function.

Single-use gloves were used to collect rectal fecal samples from each calf once a week starting at birth, prior to the first feeding in the trial (pre-treatment), until 6 weeks of age. Fecal samples were stored at −20 °C until DNA extraction. Only samples collected pre-treatment (**S0**), and on weeks one (**S1**), three (**S3**) and six (**S6**) after the beginning of the study. Furthermore, only half of the calves enrolled in this feeding trial were selected for shotgun sequencing of fecal samples, rendering a total of 14 calves, seven from each treatment group (DR and NR).

### DNA Extraction

Stored fecal samples were thawed and homogenized by vortexing for 3 minutes. A 50 mg sample was used for DNA extraction using a PowerSoil DNA Isolation Kit (MO BIO Laboratory Inc.) following the manufacturer’s recommendation. DNA concentration was evaluated using the Quant-iT PicoGreen dsDNA Assay Kit (Life Technologies Corporation,Carlsbad, CA, USA).

### Library preparation and sequencing

An aliquot of each DNA sample was normalized to 0.2 ng/μl. Once the normalization was finished the library was prepared using the Nextera XT DNA Sample Preparation Kit (Illumina Inc. San Diego, CA). Tagmentation of samples was done using 1 ng of template, as directed by manufacturer. Following tagmentation, PCR amplification was done according to manufacturer’s instructions using a unique combination of barcode primers (provided by the manufacturer) for each of the 56 samples, allowing samples to be multiplexed. Following amplification, short DNA fragments were removed using the AMPure XP bead purification kit (Beckman Coulter, Indianapolis, IN) and normalized through Library Normalization beads/additives. DNA fragments were then paired-end sequenced (250 base pair [bp] reads) in multiplexed pools on the Illumina HiSeq. 2500 platform (Illumina Inc.) at the Biotechnology Resource Center at Cornell University (Ithaca, NY).

### Bioinformatics and Statistical Analysis

Fastq format files were uploaded to the Metagenome Rapid Annotation using Subsystem Technology (MG-RAST) server. In MG-RAST, sequences were subjected to quality control, which includes dereplication (remove artificial sequences produced by sequencing artifacts), removing host specific species sequences (B. Taurus, UMD v3.0), ambiguous base filtering (removing sequences with >5 ambiguous base pairs) and length filtering (removing sequences with a length of >2 standard deviation from the mean). Of the sequences that passed these quality tests, predicted protein coding regions were assigned annotations based on the SEED sequence identification, and assigned to a functional category using the SEED subsystem.

Relative abundances of different bacterial taxa in each sample were used as covariates in stepwise discriminant analysis models built in JMP Pro 12.0.1. Each variable was removed in a stepwise manner until only variables with a *P*-value < 0.001 were retained in the final model. A series of multivariable screening analyses using JMP Pro 12.0.1 was performed to determine which bacterial species were most important to differentiate between DR and NR groups. False discovery rate (FDR) was used to correct for multiple comparisons in screening analysis. Given the large sample size, a strict cutoff (FDR-probability ≤ 0.001) was used to minimize Type-1 statistical errors.

Multiple linear regression analysis conducted in JMP 12.0.1 was used to compare the abundance of microbial functions between treatment groups for each sampling week. The dependent variable was the microbial variable being analyzed and the independent variables milk feeding treatment (DR and NR), time in weeks of sampling (S1, S3 and S6), and interactions were included in all models. The effect of repetitive sampling from individual calves and trial number was controlled in the models as a random effect. Because of its pertinence to the study topic, resistance to antibiotics and toxic compounds (RATC) functions where also further analyzed to evaluate changes between sampling time points (S0, S1, S3 and S6), and Tukey Kramer HSD was used in JMP to further evaluate significant changes in relative abundance for each sampling point.

The relative abundance of different gene functions were used as covariates in stepwise discriminant analysis models built in JMP Pro 12.0.1. In the discriminant analysis, genes were removed in a stepwise manner until only variables with a *P*-value < 0.05 were retained in the final model. Multiple discriminant analyses were conducted using the following variables as covariates: time of sampling (in weeks) by treatment group, treatment group by each week of sampling, and treatment group for all samples collected from calves after receiving the first feeding treatment (weeks 2, 4 and 7). Canonical scores for these analyses were used to create graphical display of the results.

Gene function variables were retained after the stepwise manner discriminant analysis for treatment group by discriminating or not by each week of sampling and evaluated in JMP through a screening analysis using the false discovery rate (FDR) to correct for multiple comparisons. Taxa with a significantly different relative frequency between treatment groups were selected for further analysis using a multivariate mixed logistic regression model. The independent variables treatment group, time in weeks of sampling, and interactions were included in all models. Shannon diversity indices were used to estimate diversity at the species level. The effect of animal identification nested within trial number was controlled in the models as a random effect. Least square means and standard error of the means for these indexes were obtained using the LSMEANS statement. A heatmap using the distribution of gene functions was produced using MG-Rast.

### Data Availability

The datasets used and/or analyzed during the current study are available from the corresponding author on reasonable request.

### Ethical Approval

Fecal samples were collected from calves (*Bos taurus*) that were housed on Cornell University facilities. The research protocol was reviewed and approved by the Institutional Animal Care and Use Committee of Cornell University (Protocol number: 2012–0090).

## Electronic supplementary material

Supplementary Table S1
