# Supplementary material for: Impacts of feeding preweaned calves milk containing drug residues on the functional profile of the fecal microbiota
Source: Sci Rep. 2018 Jan 11;8:554. doi: 10.1038/s41598-017-19021-2 (PMC5764986; doi:10.1038/s41598-017-19021-2)
Supplement: Supplementary file 1 — Supplementary Table S1 [file 41598_2017_19021_MOESM1_ESM.pdf]

TITLE PAGE

Impacts of feeding preweaned calves milk containing drug residues on the functional profile of the fecal microbiota

Richard Van Vleck Pereira[1]*, Laura M. Carroll [2], Svetlana Lima[3], Carla Foditsch[3], Julie D. Siler[3], Rodrigo Carvalho Bicalho[3], Lorin D. Warnick[3]

1 Department of Population Health and Reproduction; 2 Department of Food Science, Cornell University, Ithaca, New York, USA; 3 College of Veterinary Medicine, University of California Davis, Davis, CA, United States of America; Department of Population Medicine and Diagnostic Sciences. College of Veterinary Medicine, Cornell University, Ithaca, NY, United States of America. *rvpereira@ucdavis.edu

Supplementary Table S1. Summary of sequencing data by sample identification number in MG-Rast, treatment group, and week fecal sample was collected.

Sample Identification[1]	Treatment Group	Week[2]	Total Sequences (bp)	Sequences Read	Sequence Length (bp)
**4662405.3**	DR	0	1,121,759,435	4,234,859	264.9
**4662426.3**	DR	0	1,707,010,647	6,494,358	262.8
**4662409.3**	DR	0	813,918,021	2,920,080	278.7
**4662439.3**	DR	0	1,371,690,992	5,351,376	256.3
**4662407.3**	DR	0	2,301,273,093	8,606,389	267.4
**4662427.3**	DR	0	1,931,723,507	7,365,360	262.3
**4662449.3**	DR	0	253,623,749	945,411	268.3
**4662431.3**	DR	2	1,303,836,558	4,854,585	268.6
**4662447.3**	DR	2	444,353,428	1,636,126	271.6
**4662404.3**	DR	2	666,063,972	2,380,467	279.8
**4662429.3**	DR	2	396,865,509	1,499,760	264.6
**4662440.3**	DR	2	208,034,992	759,028	274.1
**4662454.3**	DR	2	192,043,366	726,065	264.5
**4668226.3**	DR	2	198,927,351	748,807	265.7
**4662434.3**	DR	4	1,617,269,807	5,981,099	270.4
**4662450.3**	DR	4	764,756,336	2,797,804	273.3
**4662414.3**	DR	4	1,334,959,205	4,862,486	274.5
**4662445.3**	DR	4	1,255,233,474	4,882,770	257.1
**4662088.3**	DR	4	761,885,164	2,899,064	262.8
**4662410.3**	DR	4	1,454,236,080	5,600,184	259.7
**4662416.3**	DR	4	439,510,331	1,583,350	277.6
**4662448.3**	DR	7	710,303,487	2,689,321	264.1
**4662430.3**	DR	7	555,300,117	2,076,205	267.5
**4662442.3**	DR	7	991,167,024	3,750,457	264.3
**4662406.3**	DR	7	1,161,081,879	4,384,049	264.8
**4662444.3**	DR	7	1,263,288,268	4,390,577	287.7
**4662453.3**	DR	7	1,547,497,877	5,522,966	280.2
**4662415.3**	NR	0	1,532,144,514	5,531,411	277.0
**4662421.3**	NR	0	392,784,698	1,448,148	271.2
**4662436.3**	NR	0	1,251,570,173	4,664,102	268.3
**4662412.3**	NR	0	1,001,446,905	3,764,573	266.0
**4662432.3**	NR	0	539,069,680	2,076,785	259.6
**4662443.3**	NR	0	489,092,636	1,905,291	256.7
**4662451.3**	NR	0	276,228,557	1,025,324	269.4
**4662417.3**	NR	2	372,607,127	1,345,719	276.9
**4662446.3**	NR	2	219,513,116	816,691	268.8

**4662419.3**	NR	2	268,748,150	1,026,993	261.7
**4662424.3**	NR	2	882,380,702	3,285,230	268.6
**4662425.3**	NR	2	1,038,860,457	3,871,199	268.4
**4662441.3**	NR	2	628,233,212	2,403,337	261.4
**4662455.3**	NR	2	333,745,121	1,244,334	268.2
**4662411.3**	NR	4	652,399,095	2,371,858	275.1
**4662418.3**	NR	4	732,565,641	2,579,448	284.0
**4662420.3**	NR	4	1,183,060,530	4,384,525	269.8
**4662408.3**	NR	4	1,978,165,892	6,957,238	284.3
**4662422.3**	NR	4	1,198,368,927	4,389,277	273.0
**4662428.3**	NR	4	1,329,145,552	4,713,367	282.0
**4668225.3**	NR	4	956,514,398	3,460,074	276.4
**4662433.3**	NR	7	1,144,404,553	4,435,861	258.0
**4662456.3**	NR	7	1,932,430,722	7,495,197	257.8
**4662423.3**	NR	7	737,507,757	2,874,828	256.5
**4662413.3**	NR	7	3,172,379,212	12,374,163	256.4
**4662435.3**	NR	7	1,939,456,377	7,531,547	257.5
**4662437.3**	NR	7	1,922,249,424	7,275,347	264.2
**4662438.3**	NR	7	1,910,316,785	7,376,622	259.0

1. Sequences were deposited in the MG-Rast database; 2.Weekly fecal sample was collected form calves after enrollment in the study. Week 0 represent a fecal sample collected at enrollment, before calves received and milk from either treatment.
